# Comparison of Measurement of Central Corneal Thickness with Spectral Domain Optical Coherence Tomography and Standard Ultrasonic Pachymeter in Premature Infants

**DOI:** 10.1155/2015/129269

**Published:** 2015-07-22

**Authors:** Emre Hekimoglu, Muhammet Kazım Erol, Devrim Toslak, Deniz Turgut Coban, Berna Doğan, Ozgur Yucel

**Affiliations:** ^1^Ophthalmology Department, Etlik Zubeyde Hanim Maternity and Women's Health Research Hospital, Yeni Etlik Caddesi No:55, Etlik, Keçiören, 06010 Ankara, Turkey; ^2^Ophthalmology Department, Antalya Training and Research Hospital, Varlık Mah., Kazım Karabekir Caddesi, Muratpaşa, 07100 Antalya, Turkey

## Abstract

*Purpose*. To evaluate the repeatability of measurement of central corneal thickness (CCT) by spectral domain optical coherence (SD-OCT) in premature infants and compare it to CCT measurement by ultrasonic pachymetry (USP).* Methods*. Three CCT measurements of the left eyes of 50 premature infants were obtained by SD-OCT using the iVue system. 10 CCT measurements of each 28 left eyes of 28 infants were obtained by USP using the Pacscan 300P system. Bland-Altman plots were developed and the limit of agreement (LoA) was determined to compare the mean of the SD-OCT and USP measurements.* Results*. No statistically significant difference was found among the 3 CCT measurements by SD-OCT. Both USP and SD-OCT have been performed for only left eyes of 28 of the 50 babies. Those results have been compared with each other. A statistically significant difference was found between the mean CCT measurements by SD-OCT and USP (*p* < 0.05). The LoA between the SD-OCT and USP measurements ranged from 11.4 to −64.1.* Conclusions*. CCT can be measured using the iVue SD-OCT system with a high level of repeatability. Although measurement of CCT by SD-OCT and USP is highly correlated, the 2 systems cannot be used interchangeably in premature infants.

## 1. Introduction

Accurate and reliable measurement of corneal thickness is clinically essential to evaluate endothelial function and ensure precise measurement of eye pressure [1]. Measurement of central corneal thickness (CCT) has been an important aspect of diagnosing and treating many diseases for a number of years [2]. Previous research has found that CCT is significantly thicker in both premature infants and full-term newborns compared to adults. CCT has also been found to be significantly thicker in premature infants compared to full-term infants, indicating that CCT declines with increasing birth weight [3].

Various methodologies based on either ultrasonic or optical principles can be used to measure CCT [4]. While the most common technique is ultrasonic pachymetry (USP) [1], its use poses several disadvantages that can cause inaccurate measurement. First, its accuracy depends on ensuring the correct localization and directionality of the probe on the cornea. Second, changes in the speed of the ultrasound waves through the cornea as they pass through tissues of varying hydration may decrease the accuracy of the measurement [5]. Finally, the need to make contact with the probe poses the risk of injury to the cornea and/or of infection [6].

To overcome these disadvantages, a number of noncontact systems have been introduced to measure corneal thickness, including the rotating Scheimpflug camera, optical coherence tomography (OCT), scanning slit topography, and specular microscopy [2]. However, none of these techniques can be used with premature infants due to this population's lack of visual fixation and the difficulty of positioning the infants correctly.

The iVue SD-OCT system (Optovue Inc., Fremont, CA, USA) is an optical technique for measuring CCT that may be appropriate for use with infants. A relatively new system based on spectral domain optical coherence (SD-OCT) technology, the iVue SD-OCT system, was first developed for posterior segment imaging. With the addition of software and the connection of external attachments with lenses, the iVue can obtain high-definition cross-sectional images with which to calculate the thickness of the central and regional cornea. Because of its portability, the iVue can overcome the positional difficulties faced when measuring the CCT in infants, allowing the user to obtain corneal images in several seconds. However, the accuracy of the measurements obtained using the iVue as a representative SD-OCT system is unclear. To address this concern, this study evaluated the repeatability of the CCT and peripheral corneal thickness measurements obtained by SD-OCT in premature infants and compared them with measurements obtained by USP.

## 2. Materials and Methods

The study was approved by the local ethics committee and performed in accordance with the ethical standards outlined in the Declaration of Helsinki. Informed consent was obtained by the parents or guardians of all participating subjects prior to their inclusion in the study. Randomly selected fifty infants who were being followed up by the retinopathy of prematurity (ROP) screening program conducted by our hospital between December 2014 and February 2015 were enrolled in this study for measurement of the left eye. The exclusion criteria were anterior segment anomalies, such as congenital glaucoma or Peters anomaly, and any retinal diseases except ROP. Before ROP examination, the iVue SD-OCT system with the Corneal Adaptor Module (CAM) version 3.1.0.17 was used to obtain a corneal pachymetry map. This device works at 830 nm, a near-infrared wavelength, and is able to obtain 26000 axial scans per second of the tissue. The CAM consists of 2 adapter lenses, a wide-angle lens and a high-magnification lens, and software. The wide-angle lens, which was used in this study, is able to obtain a scan width of 6.0 mm and a transverse resolution (focused spot size) of 15 *μ*m, while the corneal module provides corneal mapping by telecentric scanning of the anterior segment. The use of this system and the device software allows for the performance of 8 high-definition meridional scans in only 0.32 seconds and automatic detection of the anterior and posterior corneal boundaries.

**Figure 1:**
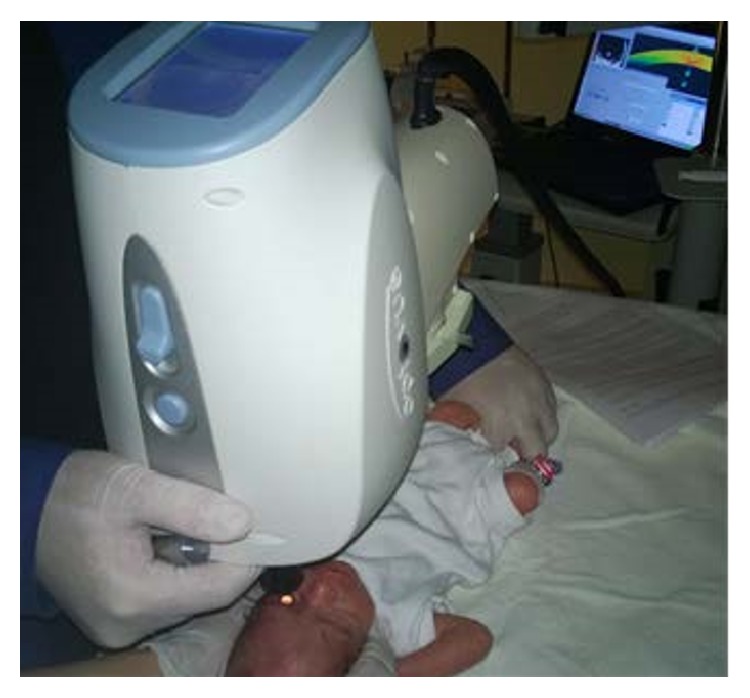
Measurement of CCT by SD-OCT in a premature infant.

The use of the iStand (Optovue Inc.), a rolling floor stand option for the iVue SDOCT system, allows patients to be scanned in various positions, including the supine position. Before examination the iVue system was mounted to the iStand to obtain images from the infant in the supine position. A drop of 0.5% proparacaine HCl solution (Alcaine; Alcon Laboratories, Fort Worth, TX, USA) was applied for topical anesthesia. No additional anesthetic or sedative drug was used. If needed, a pacifier dipped in 30% dextrose was used to calm the infant. A lid speculum was used to hold the eyelids open. A lubricant eye drop (Systane, Alcon Pharmaceuticals Ltd., Barcelona, Spain) was instilled for hydrating the cornea as necessary. During scanning, the hands of the supine-positioned infant were held by a nurse (Figure 1). Using the iVue system in the corneal pachymetry mode, in which the pupil is in the center of the small diameter circle at the screen of the computer connected to the device, three different measurements were obtained from the left eye. If the image quality was less than the manufacturer's suggested quality and/or the pupil was not centralized, the measurement was repeated after the infant had been repositioned.

For the 22 infants who appeared agitated subsequent to standard ROP examination, the examination was terminated. For the 28 infants who were sufficiently calm, the lid speculum was not removed and the supine position was maintained. Without using additional topical anesthetic drops and maintaining the supine position, 10 different measurements were taken of the 28 infants using the Pacscan 300P USP device (Sonomed Inc., Lake Success, NY, USA). The ultrasonic velocity was set at 1636 m/s in the pachymetry mode and the measurements were taken by a 45° angled probe that had been centered perpendicular to the corneal surface. The mean of the 10 measurements was calculated to perform the statistical analysis.

To evaluate the repeatability of measurements taken by SD-OCT, the 3 measurements obtained from all 50 patients using the iVue system were compared among themselves. To compare the accuracy of the SD-OCT and USP systems, the mean CCT values of the 28 patients who had been measured using both systems were compared. Statistical analysis was performed using SPSS version 20 (SPSS lnc., Chicago, IL, USA) and MedCalc version 9.6.2.0 (MedCalc Software, Mariakerke, Belgium) software. Only the data obtained from the left eye were used for statistical analysis. Descriptive statistics presented terms of the mean +/− standard deviation. Analysis of variance (ANOVA) was performed and Bland-Altman plots were developed to compare the mean of the SD-OCT measurements and the mean of the USP measurements. Intraexaminer repeatability was determined using intraclass correlation coefficients (ICCs). *p* values of 0.05 or less were considered statistically significant.

## 3. Results

**Figure 2:**
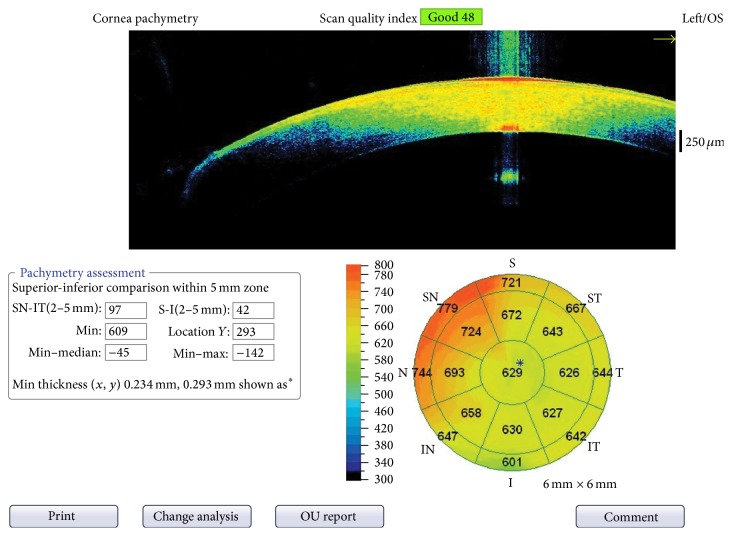
Printout of SD-OCT results showing measurement of CCT in three circles. The measurement in the innermost circle (2 mm) was accepted as the CCT value.

Of the 50 infants (28 females and 22 males) who participated in the study, SD-OCT measurements were able to be obtained from all 50 and USP measurements from 28. The mean gestational age of all 50 infants at birth was 30.5 ± 2.8 weeks (range 24–36 weeks) and the mean birth weight was 1507 ± 491 g. At measurement, the mean postmenstrual age (PMA) of all 50 infants was 39.7 ± 5.8 weeks and the mean weight was 3163 ± 1079 g. At measurement, the mean PMA of the 28 infants from whom USP measurements were obtained was 37.9 ± 3.7 weeks and the mean weight was 2926 ± 853. The mean CCT of the left eye of the 50 infants measured with SD-OCT was 541 ± 50 *μ*m in the first measurement, 539 ± 46 *μ*m in the second measurement, and 538 ± 51 *μ*m in the third measurement (Figure 2 and Table 1). No significant difference was found among the three measurements obtained by SD-OCT. In contrast, a significant difference was found between the mean of the measurement of 28 infants obtained by USP, which was 576 ± 69 *μ*m, and the mean of the three measurements, which was 549 ± 56 *μ*m, of the same 28 infants obtained by SDOCT (*p* < 0.05).

**Figure 3:**
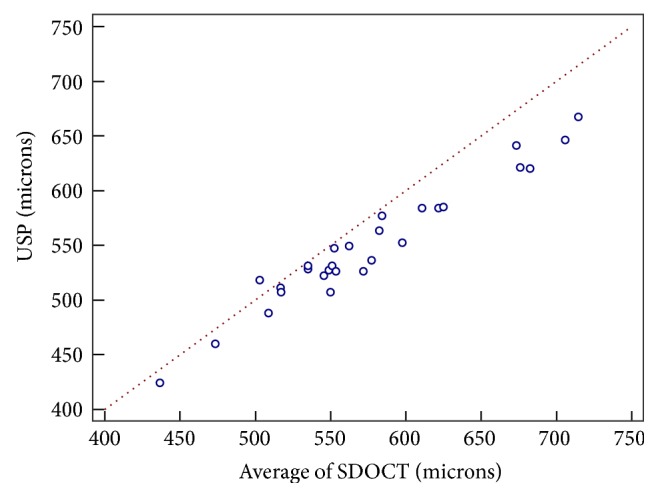
Scatter plot of correlation between mean SD-OCT and USP measurements.

**Figure 4:**
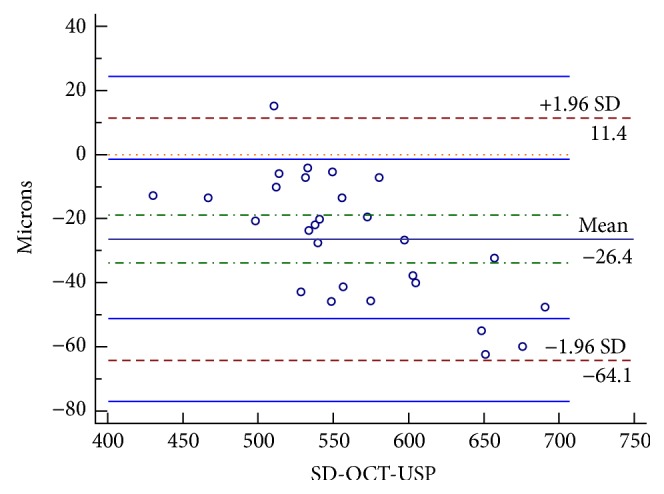
Bland-Altman plot comparing measurement of CCT by SD-OCT and USP.

**Figure 5:**
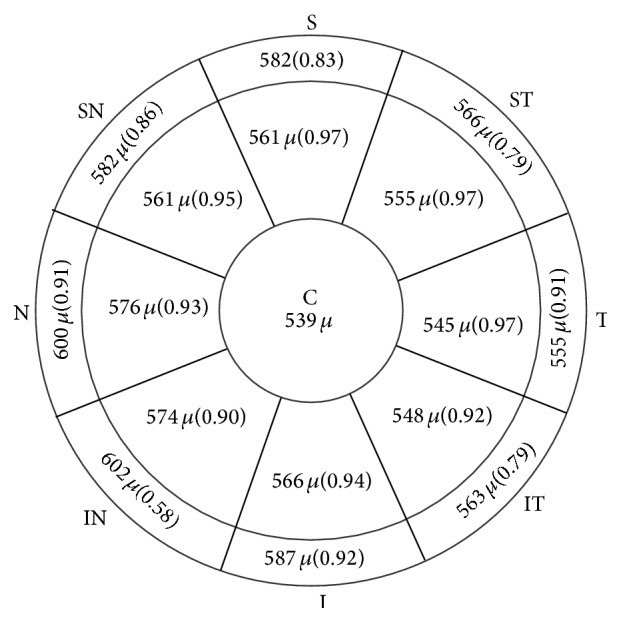
A figure showing peripheral corneal thickness in the 2–5 mm and 5-6 mm zones. The ICC values are shown after the measurements in parentheses.

Figure 3 and Table 2 show the results of the comparison of the mean of the 3 measurements obtained using SD-OCT with the measurement obtained using USP. As can be observed, development of a Bland-Altman plot revealed the limit of agreement (LoA) between the two methods to range from 11.4 to −64.1 and the mean difference between the two methods to be −26.4 *μ*m. As shown in Figures 4 and 5 and Tables 3 and 4, the intraclass correlation (ICC) values for peripheral corneal thickness were lower than the ICC values for CCT. In particular, in nasal zone between 5 and 6 mm, ICC values were the lowest (0.273–0.769).

## 4. Discussion

To our knowledge, this was the first study of the measurement of CCT measurement using SD-OCT in premature infants. Of the few studies that previously measured CCT in premature infants, USP had been used in all. Using USP, Kirwan et al. reported a mean CCT of 564 *μ*m at a PMA of 39 to 41 weeks [7], Muslubas et al. a mean of 600 *μ*m (range 515–790) at a mean PMA of 36.3 ± 0.9 weeks [8], and de Silva et al. a mean of 604 *μ*m at a mean PMA of 36.3 weeks [9]. In our previous study, we reported a mean CCT of 559 ± 42 *μ*m at a mean PMA of 40 ± 4.8 weeks [10]. This value agrees with the value we obtained using USP in the current study, which was a mean CCT of 576 ± 69 *μ*m at a mean PMA of 37.9 ± 3.7 weeks. Our findings thus agree with previous studies when the decrease in CCT from birth to term-equivalent age in premature infants is taken into account.

In the current study we found that pachymetry measurements obtained using SD-OCT are highly repeatable and correlated with the values obtained using USP, which is considered a common method for performing CCT measurement. In our previous study we found that the central foveal thickness of premature infants can be measured using the iVue SD-OCT device, a compact version of the RTVue device (Optovue Inc.), connected to the iStand, which makes the system suitable for imaging infant eyes in the supine position [11]. Similar studies including adult subjects also found the measurements obtained using the RTVue device to be highly repeatable. In fact, some even reported that the CCT measurements obtained by RTVue were more highly repeatable than those obtained by other commonly used devices, such as devices used to conduct USP and corneal topography [2, 6].

We found that ICC values for peripheral corneal thickness were lower than those for CCT. This finding is predictable considering the steeper cornea of infants compared to adults [12] and that the reflection in the central cornea is strong but decreases peripherally, which can result in inaccurate measurement, especially when measuring steep corneas. As similar studies had, we found a high correlation between the values obtained by SD-OCT and USP. However, we found a wide range of LoA values using the Bland-Altman method. Studies of CCT measurements of adults using the RTVue device have reported lower values than those obtained by USP as well as SD-OCT measurements that were 5.6 to 18 *μ*m lower than USP measurements [1, 4]. This difference has been attributed to the decentralization or placement of the ultrasonic probe obliquely rather than perpendicularly or of variation in the sound speed according to tissue hydration.

Central corneal hydration has an important role in determining corneal thickness in premature babies. Because of the improved hydration control it is thought that thickness of cornea decreases after birth. It is suggested that growth of the eye, with possible remodelling and stretching of collagen fibres, plays a significant role in corneal development after birth [7].

Bradfield et al. showed that the range of CCT measurements by the USP at each age (from birth to 17 years old) was approximately 120 *μ*m and they noticed that the clinical significance of this range is unknown [13].

In our study of infants, we found the difference in the measurements obtained by SD-OCT and USP (26 *μ*m) to be more than that obtained in the adult studies. We attribute this difference in findings to the thicker and more hydrated corneal structure of the premature infant and/or the involuntary movement of the infant during the imaging session. Although a previous study of children from birth to age 17 reported that the reliability of USP was not influenced by age, race, ethnicity, or examination setting, CCT measurement has been found less reliable for thicker corneas. Based on this finding, Weise et al. concluded that the measurement should be repeated when the CCT is thicker than 575 *μ*m [14].

Uva et al. showed that the main factor affecting intraocular pressure (IOP) is CCT [3]. However, CCT has been found to influence measurement by the Tono-Pen (Reichert Technologies, Depew, NY, USA) less than that by other tonometers [15]. A study of 2079 children aged 0 to 17 years using handheld USP and Tono-Pen found that IOP increased 1.5 mmHg in every 100 *μ*m of CCT [16]. Likewise, Burdová et al. reported that IOP increased 2 mmHg and 2.5 mmHg in every 100 *μ*m of CCT when using the Tono-Pen and the applanation tonometer, respectively [17]. Consideration of these findings indicates that a 50 *μ*m change in corneal thickness may result in an IOP change of 1 mmHg. According to this indication, the mean 26.4 *μ*m lower CCT measurement obtained with SD-OCT than with USP equates to a less than 1 mmHg decrease in IOP in premature infants.

We propose that not only can SD-OCT be substituted for USP but that it also has the advantage of evaluating the corneal layers while measuring the corneal thickness. Thus, it allows for structural evaluation of the cornea in the presence of any corneal pathology, such as corneal infection or ulcer and congenital anterior segment anomalies, such as Peters anomalies, and/or persistent tunica vasculosa lentis. Additionally, the iris and the anterior surface of the lens capsule could be observed and the iridocorneal angle could be measured with SD-OCT. As a noncontact method, SD-OCT provides the additional advantage of preventing corneal injury and infection in premature infants.

This study faced a limitation that should be considered when reviewing the findings. The corneal vertex and the pupil are 2 landmarks used to center the cornea during CCT measurement by SD-OCT. Because of the dilation of the pupil and the involuntary movement of the infant, it was difficult to center the cornea, and thus the CCT values may have deviated more than they would have in adult subjects. Despite this limitation, the findings of our study indicate that measurement of CCT using the iVue SD-OCT system has a level of repeatability. Although we found the measurements by SD-OCT to be highly correlated with those taken with US pachymetry, they did not reach a sufficiently high level of correlation to allow us to conclude that SD-OCT and USP can be used interchangeably in premature infants.

## Figures and Tables

**Table 1:** Comparison of 3 measurements by SD-OCT in 50 premature infants.

	*N*	Mean CCT	Std. dev.	95% confidence interval (lower–upper)	Min	Max
SD-OCT 1	50	541	50	526–555	470	723
SD-OCT 2	50	539	46	526–552	473	723
SD-OCT 3	50	538	51	524–553	465	726

**Table 2:** Comparison of 3 measurements by SD-OCT in 28 premature infants and mean of 10 measurements by USP.

	*N*	Mean	Std. dev.	95% confidence interval (lower–upper)		*p* value
SD-OCT 1	28	552	58	510	584	
SD-OCT 2	28	549	58	507	581	
SD-OCT 3	28	548	57	506	580	
USP	28	576	69	528	603	<0.001

**Table 3:** Pairwise comparison of ICC values and confidence intervals.

Measurements compared	ICC (95% CI)	*p* value
SD-OCT 1 and SD-OCT 2	0.984 (0.974–0.991)	*p* < 0.001
SD-OCT 1 and SD-OCT 3	0.984 (0.969–0.992)	*p* < 0.001
SD-OCT 2 and SD-OCT 3	0.985 (0.971–0.992)	*p* < 0.001
USP and SD-OCT 1	0.960 (0.918–0.980)	*p* < 0.001
USP and SD-OCT 2	0.976 (0.947–0.989)	*p* < 0.001
USP and SD-OCT 3	0.976 (0.928–0.992)	*p* < 0.001

**Table 4:** Repeatability (ICC value) of peripheral corneal thickness measurement according to region.

Region	ICC (95% CI)	*p* value	Mean	Min–max
Inferior 2–5 mm	0.944 (0.903–0.969)	*p* < 0.001	567	564–580
Inferior 5-6 mm	0.927 (0.874–0.960)	*p* < 0.001	588	584–592
Inferior temporal 2–5 mm	0.920 (0.862–0.956)	*p* < 0.001	549	542–555
Inferior temporal 5-6 mm	0.793 (0.643–0.886)	*p* < 0.001	563	559–566
Temporal 2–5 mm	0.972 (0.952–0.985)	*p* < 0.001	546	541–548
Temporal 5-6 mm	0.914 (0.852–0.953)	*p* < 0.001	556	553–560
Superior temporal 2–5 mm	0.973 (0.953–0.985)	*p* < 0.001	556	552–559
Superior temporal 5-6 mm	0.792 (0.641–0.886)	*p* < 0.001	566	561–573
Superior 2–5 mm	0.970 (0.947–0.983)	*p* < 0.001	562	559–564
Superior 5-6 mm	0.827 (0.701–0.905)	*p* < 0.001	582	578–588
Superior nasal 2–5 mm	0.948 (0.911–0.972)	*p* < 0.001	562	559–561
Superior nasal 5-6 mm	0.860 (0.759–0.923)	*p* < 0.001	583	574–591
Nasal 2–5 mm	0.930 (0.879–0.961)	*p* < 0.001	576	571–579
Nasal 5-6 mm	0.908 (0.841–0.949)	*p* < 0.001	601	589–610
Inferior nasal 2–5 mm	0.900 (0.827–0.945)	*p* < 0.001	574	562–586
Inferior nasal 5-6 mm	0.578 (0.273–0.769)	*p* < 0.001	602	594–609

## References

[1] Chen S., Huang J., Wen D., Chen W., Huang D., Wang Q. (2012). Measurement of central corneal thickness by high-resolution Scheimpflug imaging, Fourier-domain optical coherence tomography and ultrasound pachymetry. *Acta Ophthalmologica*.

[2] Ishibazawa A., Igarashi S., Hanada K. (2011). Central corneal thickness measurements with Fourier-domain optical coherence tomography versus ultrasonic pachymetry and rotating scheimpflug camera. *Cornea*.

[3] Uva M. G., Reibaldi M., Longo A. (2011). Intraocular pressure and central corneal thickness in premature and full-term newborns. *Journal of AAPOS*.

[4] Bayhan H. A., Bayhan S. A., Can I. (2014). Comparison of central corneal thickness measurements with three new optical devices and a standard ultrasonic pachymeter. *International Journal of Ophthalmology*.

[5] González-Méijome J. M., Cerviño A., Yebra-Pimentel E., Parafita M. A. (2003). Central and peripheral corneal thickness measurement with Orbscan II and topographical ultrasound pachymetry. *Journal of Cataract and Refractive Surgery*.

[6] Rao H. L., Kumar A. U., Chary S., Senthil S., Vaddavalli P. K., Garudadri C. S. (2011). Evaluation of central corneal thickness measurement with RTVue spectral domain optical coherence tomography in normal subjects. *Cornea*.

[7] Kirwan C., O'Keefe M., Fitzsimon S. (2005). Central corneal thickness and corneal diameter in premature infants. *Acta Ophthalmologica Scandinavica*.

[8] Muslubas I. B. S., Oral A. Y. A., Cabi C., Caliskan S. (2014). Assessment of the central corneal thickness and intraocular pressure in premature and full-term newborns. *Indian Journal of Ophthalmology*.

[9] de Silva S., Parentin F., Michieletto P., Pensiero S. (2011). Corneal curvature and thickness development in premature infants. *Journal of Pediatric Ophthalmology and Strabismus*.

[10] Ozdemir O., Tunay Z. Ö., Petriçli I. S., Acar D. E., Acar U., Erol M. K. (2014). Analysis of the horizontal corneal diameter, central corneal thickness, and axial length in premature infants. *Arquivos Brasileiros de Oftalmologia*.

[11] Erol M. K., Ozdemir O., Turgut Coban D. (2014). Macular findings obtained by spectral domain optical coherence tomography in retinopathy of prematurity. *Journal of Ophthalmology*.

[12] Inagaki Y. (1986). The rapid change of corneal curvature in the neonatal period and infancy. *Archives of Ophthalmology*.

[13] Bradfield Y. S., Melia B. M., Repka M. X. (2011). Central corneal thickness in children. *Archives of Ophthalmology*.

[14] Weise K. K., Kaminski B., Melia M. (2013). Intraobserver reliability of contact pachymetry in children. *Journal of AAPOS*.

[15] Sahin A., Basmak H., Yildirim N. (2008). The influence of central corneal thickness and corneal curvature on intraocular pressure measured by tono-pen and rebound tonometer in children. *Journal of Glaucoma*.

[16] Bradfield Y. S., Melia B. M., Repka M. X. (2011). Central corneal thickness in children. *Archives of Ophthalmology*.

[17] Burdová M. C., Ferrová K., Filouš A., Oskorypová K., Ležatková P., Sedláčková P. (2011). Correlation of intraocular pressure measured by applanation tonometry, noncontact tonometry and tonopen with central corneal thickness. *Ceska a Slovenska Oftalmologie*.

